# Editorial: Blood vessel management in surgical oncology

**DOI:** 10.3389/fonc.2023.1180216

**Published:** 2023-03-20

**Authors:** Ulrich Ronellenfitsch, Jens Jakob

**Affiliations:** ^1^ Department of Abdominal, Vascular and Endocrine Surgery, Martin-Luther-University Halle-Wittenberg, University Medical Center Halle (Saale), Halle (Saale), Germany; ^2^ Sarcoma Unit, Department of Surgery and Mannheim Cancer Center, University Medical Center Mannheim, University of Heidelberg, Mannheim, Germany

**Keywords:** surgical oncology, vascular surgery, blood vessels, anatomy, resections

The management of blood vessels is of high importance in many oncological resections. Malignant tumors with an invasion of adjacent arteries or veins or tumors of vascular origin require resection of these blood vessels, and, depending on the specific vessel, vascular reconstruction to ensure sufficient end-organ perfusion or venous drainage (1, 2). In tumors that abut vessels, but do not infiltrate them and do not show circumferential growth, such as for example pancreatic carcinomas, periadventitial dissection, sometimes named “divestment”, can be sufficient (3). In tumors for which adequate oncological resection comprises systematic lymphadenectomy, such as colorectal cancers, lymph node harvesting can be guided by vascular structures (4). Decisions when such techniques should be employed require broad knowledge and expertise of both the oncological characteristics of the underlying disease and of surgical techniques, possibilities and limitations.

This Research Topic provides an evidence-based overview on a number of aspects of blood vessel management in surgical oncology. Retroperitoneal tumors, which comprise sarcoma, renal cell, and adrenal carcinoma, and pheochromocytoma, frequently affect the renal veins and inferior vena cava (IVC), possibly causing tumor thrombosis (5). Liu et al. present a series of 19 resected patients with retroperitoneal tumors of various entities, which showed venous tumor thrombus. The series shows that such tumors can be resected with acceptable morbidity in experienced facilities, and that even minimally-invasive approaches are feasible in specific situations. The authors propose a grading system for retroperitoneal venous tumor thrombosis and describe dedicated surgical techniques for the different grades. A second case series from the same group describes their experience with robotic-assisted laparoscopic surgery for retroperitoneal tumors affecting the IVC (Liu et al.). It comprises 17 cases, 11 of which are renal cell carcinoma. Also in this series, operative outcomes were remarkable with very low complication rates. Of note, techniques also included IVC ligation for tumor thrombus without severe complications due to venostasis being reported by the authors.

Further caudally located sarcomas tend to affect the iliac vessels. Lv et al. illustrate their experience regarding en bloc resection with graft interposition using the abdominoinguinal approach with a series of 24 consecutive patients with retroperitoneal sarcoma. Most patients underwent iliac vein and/or artery reconstruction, and in about a third of patients, mesh reconstruction of the lower abdominal wall was done. Operative results were good with no mortality and a favorable complication rate.

Two other studies in this Research Topic deal with the vascular anatomy with regard to colectomies for colon cancer. Liao et al. describe a hitherto unnamed subpancreatic vessel, which in their series of patients who underwent colectomy for transverse or descending colon carcinoma was present in 85% of patients (Liao et al.). This vessel is of possible importance for complete mesocolic excision and lymphadenectomy, which constitute integral parts of oncological colectomies (6). Suh et al., in their study, address inferior mesenteric vein preservation during oncological left hemicolectomy. Their retrospective data points towards a lower incidence of intestinal complications in the relatively small group of patients (n=22) in whom the vein was preserved with one recurrence reported during a median follow-up of 32 months. This finding contrasts with the common recommendation to divide the inferior mesenteric vein at the lower margin of the pancreas during left hemicolectomy (7).

In summary, this Research Topic presents stimulating new findings on several aspects of blood vessel management in surgical oncology. All the presented evidence is from relatively small retrospective series, which reflects the many facets of the controversial issues and the difficulty to generalize research questions in blood vessel management to large patient collectives. Such lower-level evidence can help guiding treatment in specific situations and generating further hypothesis. Nonetheless, efforts should be made to generate higher-level evidence form prospective studies also for blood vessel management in surgical oncology.

## Author contributions

All authors have drafted the manuscript, provided intellectual content, and approved of the final version of the manuscript.
